# Probing the Structural Dynamics of the *Plasmodium falciparum* Tunneling-Fold Enzyme 6-Pyruvoyl Tetrahydropterin Synthase to Reveal Allosteric Drug Targeting Sites

**DOI:** 10.3389/fmolb.2020.575196

**Published:** 2020-09-25

**Authors:** Afrah Khairallah, Caroline J. Ross, Özlem Tastan Bishop

**Affiliations:** Research Unit in Bioinformatics (RUBi), Department of Biochemistry and Microbiology, Rhodes University, Grahamstown, South Africa

**Keywords:** *de novo* folate synthesis pathway, malaria, antifolate drugs, structural dynamics, motif discovery, allosteric modulation

## Abstract

The *de novo* folate synthesis pathway is a well-established drug target in the treatment of many infectious diseases. Antimalarial antifolate drugs have proven to be effective against malaria, however, rapid drug resistance has emerged on the two primary targeted enzymes: dihydrofolate reductase and dihydroptoreate synthase. The need to identify alternative antifolate drugs and novel metabolic targets is of imminent importance. The 6-pyruvol tetrahydropterin synthase (PTPS) enzyme belongs to the tunneling fold protein superfamily which is characterized by a distinct central tunnel/cavity. The enzyme catalyzes the second reaction step of the parasite’s *de novo* folate synthesis pathway and is responsible for the conversion of 7,8-dihydroneopterin to 6-pyruvoyl-tetrahydropterin. In this study, we examine the structural dynamics of *Plasmodium falciparum* PTPS using the anisotropic network model, to elucidate the collective motions that drive the function of the enzyme and identify potential sites for allosteric modulation of its binding properties. Based on our modal analysis, we identified key sites in the N-terminal domains and central helices which control the accessibility to the active site. Notably, the N-terminal domains were shown to regulate the open-to-closed transition of the tunnel, via a distinctive wringing motion that deformed the core of the protein. We, further, combined the dynamic analysis with motif discovery which revealed highly conserved motifs that are unique to the Plasmodium species and are located in the N-terminal domains and central helices. This provides essential structural information for the efficient design of drugs such as allosteric modulators that would have high specificity and low toxicity as they do not target the PTPS active site that is highly conserved in humans.

## Graphical Abstract

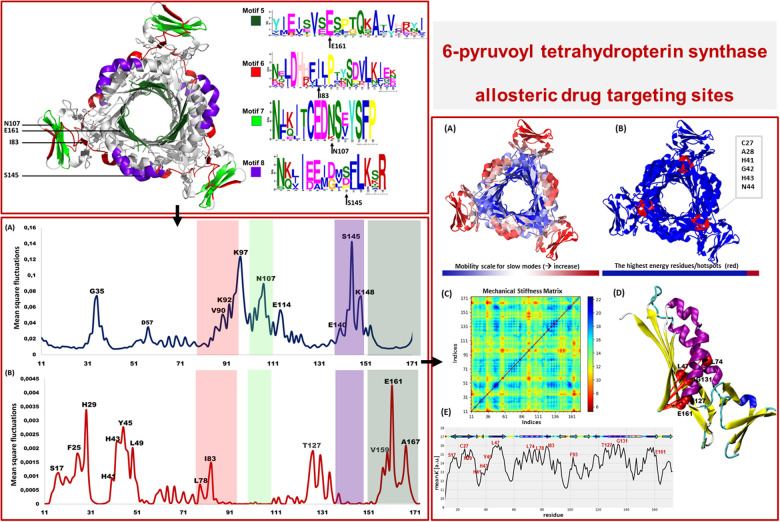

## Introduction

Despite the major progress achieved to eradicate malaria, the mosquito-borne disease remains a major public health problem (World Health Organization, 2018). Malaria is transmitted through the bite of an infected female Anopheles mosquito. Five species from the genus Plasmodium cause malaria in humans, namely: *Plasmodium falciparum (P. falciparum), P. vivax, P. ovale, P. malariae and P. knowlesi*. Among these five species, *P. falciparum* is the most pathogenic (Snow, 2015).

Tetrahydrofolate derivatives are essential for the one-carbon unit transfer during nucleotide biosynthesis and amino acid metabolism (Nzila et al., 2005). This has led to the *de novo* folate synthesis pathway being recognized as an attractive metabolic target for the treatment of numerous infectious diseases, including malaria (Swarbrick et al., 2009). The antimalarial antifolate drugs target the *de novo* folate synthesis pathway of the parasite to limit the production of folate derivatives and thereby prevent the growth and reproduction of the parasite. The two main targeted enzymes are dihydrofolate reductase (DHFR) and dihydropteroate synthase (DHPS), which are inhibited by the use of pyrimethamine and sulfadoxine antifolate drugs. The two drugs have also been used in synergy to target both enzymes simultaneously. However, resistance against the available antifolate drugs has emerged rapidly and has been observed even with the use of higher drug dosages, rendering these drugs ineffective in many cases. The combination therapy was previously used to overcome the resistance, however, in many cases, this treatment also failed as the parasite has also developed tolerance to a combination of drugs (Alker et al., 2008). The emerging resistance to antimalarial drugs drives a continuous need to develop drugs that have a novel mechanism of action. In this study, we explore an alternative drug target and drug targeting sites in the *de novo* folate synthesis pathway.

The first reaction of the *de novo* folate synthesis pathway is catalyzed by guanosine-5’-triphosphate (GTP) cyclohydrolase (GCH1), which converts the GTP moiety to the 7,8-dihydroneopterin (DHNP) (Colloc’h et al., 2000). The enzyme 6-pyruvol tetrahydropterin synthase (PTPS) then converts DHNP to 6-pyruvoyl-tetrahydropterin (PTP) via an internal redox transfer and final elimination of the DHNP phosphate tail (Bürgisser et al., 1995). The PTP product is then processed further by the downstream enzymes of the *de novo* folate synthesis pathway, including 6-hydroxymethyldihydropterin pyrophosphokinase (HPPK), DHPS, dihydrofolate synthase (DHFS) and DHFR (Kümpornsin et al., 2014).

**FIGURE 1:**
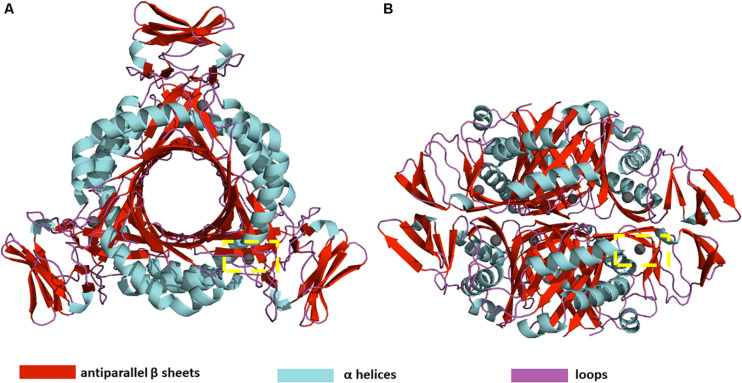
**(A)** Top view **(B)** Side view of *P. falciparum* PTPS 3D structure (PDB: 1Y13). The 3D structure is composed of a dimer of trimers with D3 symmetry (Nar et al., 1994). The yellow dashed box shows the location of one of the six active sites.

PTPS is a hexameric lyase enzyme that has 3-fold symmetry (Bürgisser et al., 1995). The protein is composed of six identical monomers that assemble via tight hydrogen bonds to form two trimer structures (Nar et al., 1994). The two trimers join via a head-to-head association to form the functional hexameric unit (Figure 1). The trimers are 60 Å in diameter and have a height of 30 Å (Nar et al., 1994). The structure of PTPS is characterized by a conically shaped central barrel that accumulates a cluster of basic and aromatic residues (Nar, 2011). PTPS has six zinc-containing active sites, and each site is buried in a deep cavity of 12 Å formed between every three adjacent monomers (Figure 1). Three histidine residues (H29, H41, H43) coordinate the metal ion through their NE2 atoms during the catalysis of the substrate (Khairallah et al., 2020). The deeply buried active sites of PTPS are accessible to the substrate through the central opening along the axis of the trimer (Bürgisser et al., 1995). Previous studies suggested that the barrel is necessary for the stabilization of the multimeric association and therefore incorporates a sophisticated use of functional allostery (Colloc’h et al., 2000). The PTPS enzyme belongs to the tunneling fold (T-fold) protein superfamily (Colloc’h et al., 2000). Enzymes of the T-fold superfamily exhibit a conserved structural topology and, with the exception of the active residues, have low sequence similarity (Colloc’h et al., 2000). The structural conservation that exists despite the low sequence similarity, points to its importance in maintaining protein function. Therefore, the characterized structural features of this protein superfamily, including its distinct central cavity, may encompass undiscovered allosteric sites that can be exploited for drug discovery.

Allostery is defined as the regulation of a protein’s structure and activity by the binding of an effector molecule at a site other than the conserved active site (Nussinov and Tsai, 2013). Allosteric drugs are highly specific to sites other than the active sites, and therefore induce the desired effect of either activating or inhibiting a protein via a mechanism that does not rely on targeting a site that is highly conserved in humans. As a result, they are considered to be far less toxic to the host. However, discovering allosteric sites that have a dominant effect on protein conformation and respective compounds that modulate these sites is far more challenging than orthosteric drug discovery (Lu et al., 2019; Amamuddy et al., 2020). As in many cases, the location of allosteric sites is often unknown and their effects on the intrinsic motion of the protein are difficult to determine experimentally (Suplatov and Švedas, 2015). Our recent review article proposes a number of integrated computational approaches to identify allosteric sites (Amamuddy et al., 2020).

Protein structures are dynamic in that they undergo large-scale domain changes in response to the binding of ligands or other compounds, thereby assuming different conformational states that allow them to perform certain functions (Bahar et al., 2007; Henzler-Wildman and Kern, 2007; Teilum et al., 2009; Nussinov and Ma, 2012; Loutchko and Flechsig, 2020; Zhang et al., 2020). Studying large-scale structural changes can elucidate key sites that modulate the functional mechanisms of a protein (Penkler et al., 2017, 2018; Guarnera and Berezovsky, 2020; Shrivastava et al., 2020). Several experimental and computational approaches are used to measure quantitatively the structure, dynamics, and function of macromolecules. A comprehensive review of different approaches is provided by Orozco (2014); Palamini et al. (2016), and Maximova et al. (2016). In this study, we have applied Normal Mode Analysis (NMA), calculated on the Anisotropic Network Model (ANM) to uncover collective motions that are essential for the tunnel gating mechanism of PTPS, and thus pinpoint potential sites that modulate the allostery of the enzyme to be targeted in future anti-malarial drug discovery. The NMA analysis was combined with protein motif discovery to elucidate motifs that are located in regions that are essential to the dynamics of PTPS and that are uniquely conserved in Plasmodium PTPS enzymes. The identified motifs further support our finding of regions responsible for the PTPS conformational transitions and therefore establish guidelines toward the selective inhibition of this enzyme.

## Materials and Methods

### Structural and Sequence Data Retrieval

The PTPS crystal structure of the *P. falciparum* was retrieved from the online Protein Data Bank (PDB ID: 1Y13). The protein functional unit consists of six identical chains, a total of 978 residues. This structure was used to construct an ENM on the C_β_ atomic coordinates of the protein, as found in the PDB file. The C_β_ atoms were selected because it has been shown that they provide a better representation of the side chains orientation. A harmonic potential within a cut-off distance (*Rc)* of 15 Å was used to account for the pairwise interactions between all of the C_β_ atoms.

For motif analysis, the protein sequences of PTPS from 20 different species, including 9 Plasmodium species, four mammalian species, four bacteria species and three fungi species (Supplementary Table S1) were retrieved using the *P. falciparum* PTPS sequence as a query in a BLAST search (Altschul et al., 1990) to identify other Plasmodium homologs in the PlasmoDB (Aurrecoechea et al., 2009) and mammalian, bacterial and fungi homologs in UniProt (Bateman, 2019). The BLAST search tool was used with default alignment parameters.

### Calculation of the Normal Modes

NMA was predominantly performed using the MODE-TASK tool suite (Ross et al., 2018) which employs the ANM (Atilgan et al., 2001) to construct an ENM of the protein structure. Although the ANM has been described previously (Atilgan et al., 2001), we provide a summary here. In the ANM, each residue of the *P. falciparum* PTPS structure was represented by a node placed at its C_β_ atom coordinate and all interactions between each pair of residues separated within a defined cutoff distance, *Rc* = 15 Å, was modeled as a set of springs with a uniform force constant γ. This yielded a network that contained *N* nodes and *M* springs representing the total number of interactions defined in the network, such that any given pair of nodes within *Rc* of each other will interact in accord with a conventional harmonic potential. The normal modes of this network are calculated in the absence of an external force, under an equilibrium condition. In the general case of *N* residues connected by *M* springs, the Hessian Matrix **H** is a 3*N* × 3*N* super-matrix that may be derived from the second derivatives of the overall potential *V*, with respect to the components of **R*_i_***, where **R*_j_*** are the fluctuation vectors of the individual residues. Therefore, the Hessian matrix describes the force constant of the system. **H** is composed of *N* × *N* super-elements, i.e.,

(1) $$H=\left[\begin{array}{l} H_{11}\;H_{12}\ldots H_{1N}\\ H21\;\ldots H_{2N}\\ \;\vdots \;\ldots \;\vdots \\ H_{N1}\;\ldots H_{NN} \end{array}\right]$$

where each super-element **H*_ij_*** is a 3 × 3 matrix that holds the anisotropic information regarding the orientation of nodes *i,j* The *ij*^th^ super-element (*i*≠*j*) of **H** is defined as:

(2) $$H_{\text{ij}}=\left[\begin{array}{ccc} \partial^{2}V/\partial X_{i}\,\partial X_{j} & \partial^{2}V/\partial X_{i}\,\partial Y_{j} & \partial^{2}V/\partial X_{i}\,\partial Z_{j}\\ \partial^{2}V/\partial Y_{i}\,\partial X_{j} & \partial^{2}V/\partial Y_{i}\,\partial Y_{j} & \partial^{2}V/\partial Y_{i}\,\partial Z_{j}\\ \partial^{2}V/\partial Z_{i}\,\partial X_{j} & \partial^{2}V/\partial Z_{i}\,\partial Y_{j} & \partial^{2}V/\partial Z_{i}\,\partial Z_{j} \end{array}\right]$$

At equilibrium, the second derivatives may be calculated for the ANM using the β-carbon position vectors of PDB structures such that the elements of the off-diagonal **H*_ij_*** are given by the equation:

(3) $$\partial^{2}V/\partial X_{i}\,\partial Y_{j}=-\gamma \,\left(X_{j}-X_{i}\right)\,\left(Y_{j}-Y_{i}\right)/S_{\mathrm{ij}}^{2}$$

And the elements of the diagonal super-elements **H_ii_** are given by the equations:

(4) $$\partial^{2}V/\partial X_{\text{i}}^{2}=\gamma \,\sum_{j}{\left(X_{j}-X_{i}\right)}^{2}/S_{\mathrm{ij}}^{2}$$

For the diagonal elements of **H_ii_** and

(5) $$\partial^{2}V/\partial X_{\text{i}}\,\partial Y_{\text{j}}=\gamma \,\sum_{j}\left(X_{j}-X_{i}\right)\,\left(Y_{j}-Y_{i}\right)/S_{\mathrm{ij}}^{2}$$

For the off-diagonal elements of **H_ii_**

**H** can be decomposed into 3*N-*6 eigenvalues and 3*N-*6 eigenvectors that correspond to the respective frequencies and directions of the individual non-trivial modes. The modes with the lowest frequencies are termed the slowest modes and define the most collective, or global, motions of the protein. The highest frequency modes describe the more localized motions of the protein. The VMD program (Humphrey et al., 1996) was used to visualize the eigenvectors describing the structural change in each mode. Lastly, mechanical stiffness calculations (Eyal and Bahar, 2008) were performed on the ANM of the PTPS using the ProDy Python package (Bakan et al., 2011, 2014) and the obtained results were visualized using Matplotlib library and VMD.

In addition, the Gaussian Network Model (GNM) calculations were performed using the DynOmics portal (Li et al., 2017), specifically to identify hinge regions within the structure of PTPS. The GNM calculations were performed using a cutoff distance of 15 Å, with a spring constant scaling factor of 10 for contact distances ≤4.0 Å and a distance scaling exponent of 2. The Dynomics portal was further used to validate the high-frequency vibrating residues and to analyze the mechanical properties of PTPS, by obtaining a color-coded representation of the PTPS structure based on the mobility of the residues from the resultant low and high GNM modes.

### Calculation of the Residue Mean Square Fluctuations

The inverse of **H** is equivalent to the covariance matrix C that is composed of *N* × *N* super-elements. Each off-diagonal, *ij*th, super-element of **H**^–1^ contains the 3 × 3 matrix of correlations between the x-, y- and z-components of fluctuation vectors of residues *i* and *j*, while the *i*th super-element of **H**^–1^ describes the self-correlation between the components of fluctuation vectors of residue *i*. For any given mode, the mean square fluctuations (MSF) of individual residues can be obtained by summing the components of fluctuation. The MSF profiles from individual normal modes were calculated using MODE-TASK (Ross et al., 2018).

### Residue Cross-Correlation Analysis

The BIO3D R package for the exploratory analysis of the structure and sequence data (Grant et al., 2006) was used to compute the deformation energy and residue cross-correlation. The BIO3D cross-correlation *S*(*i*, *j*) is given by:

(6) $$S\left(i,j\right)=\left(\Delta_{\mathrm{ri}}.\Delta_{\mathrm{rj}}\right)/{\left(\Delta r_{i}^{2}\right)}^{\frac{1}{2}}{\left(\Delta_{\mathrm{rj}}^{2}\right)}^{\frac{1}{2}}$$

where Δr*_i_* and Δr*_j_* are the displacement vectors for atoms *i* and *j*, respectively. The elements *S*(*i*, *j*) are stored in matrix form and displayed as a three-dimensional dynamical cross-correlation map. If S*ij* = 1 the fluctuations of atoms *i* and *j* are completely correlated (same period and same phase), if *Sij* = -1 the fluctuations of atoms *i* and *j* are completely anti-correlated (same period and opposite phase), and if *Sij* = 0 the fluctuations of *i* and *j* are not correlated.

The deformation energy signifies the energy density due to deformation as a function of position (Hinsen, 1998) given by Eq. 7. It provides a measurement of the individual atom’s energy contribution toward the structural deformation. PTPS deformation energy was derived from the eigen energies and vectors of the first not trivial 20 normal modes according to the equation:

(7) $$E=\frac{1}{2}\,\sum_{1}^{N}K\,\left(R_{\mathrm{ij}}^{\left(0\right)}\right)\,\frac{|\left(r_{i}-r_{j}\right).R_{\mathrm{ij}}^{\left(0\right)}{|}^{2}}{{\left|R_{\mathrm{ij}}^{\left(0\right)}\right|}^{2}}$$

where r*_i_*, r*_j_* donate the displacement of atom *i* and *j* in the mode to be analyzed, R*ij*^(0)^ is the pair distance vector (R*i*–R*j*) in the input arrangement and K is the pair force constant.

### Motif Discovery

Motif discovery was performed using Multiple Expectation Maximisation for Motif Elicitation (MEME) vs 4.11 (Bailey et al., 2015) to identify highly conserved motifs in the Plasmodium PTPS enzymes. A fasta file containing PTPS protein sequences was parsed to the MEME analysis software. The motifs search size was set to the range between 6 and 20 residues. The Motif alignment search tool (MAST) was then used to identify overlapping motifs (Bailey and Gribskov, 1998). A Python script was then used to analyze MAST files and MEME log files. Motif conservation was calculated as a number of sites per the total number of sequences, and the results were displayed as heatmaps. Motifs that were uniquely conserved in Plasmodium species were mapped onto the 3D structure of *P. falciparum* PTPS and visualized using the PyMOL Molecular Graphics System (DeLano, 2014).

## Results and Discussion

### Notable Conformational Changes Were Captured by the Lowest Frequency Non-degenerate Modes of *P. falciparum* PTPS

In this study, the ANM was applied to the structure of PTPS to classify its collective motions that have the propensity to lead the enzyme from one conformational state to another, and thus modulate its function. Further investigation of the residue fluctuations within the modes was performed to identify highly active regions that may drive these motions. The nature of allosteric modulation requires a high degree of collectivity which is often well-described by the low-frequency modes (Bahar et al., 2010). The NMA of PTPS (a 3-fold symmetry structure) yielded a total of 2,934 modes. The first 20 slowest non-trivial modes were selected for the characterization of the global intrinsic motions of the protein structure.

Normal modes obtained from symmetrical structures are highly susceptible to degeneracy, thus producing degenerate and non-degenerate modes. The degenerate modes share the same frequency and consequently any orthogonal transformation. In contrast, non-degenerate modes characterize unique directions of motions that often capture global meaningful motions that account for large conformational changes. Previous studies showed that dominant conformational changes of complexes were captured within the slowest non-degenerate modes (Atilgan et al., 2001; Chennubhotla et al., 2005; Shrivastava and Bahar, 2006; Wako and Endo, 2011; Isin et al., 2012; Lee et al., 2017; Ross et al., 2018). Here, we identified eight non-degenerate modes of PTPS. These non-degenerate modes exhibited unique eigenvalues (i.e., frequencies) and displayed unique global motions. Table 1 shows the first 20 non-trivial normal modes of PTPS, their associated frequencies, degeneracy levels and the contribution of each mode to the overall motion of the protein. Although the contribution of the individual modes was low and did not reveal a single dominant motion, we suspect that this may be a consequence of the large size of the protein and the extensive degeneracy of the normal modes. Here we have analyzed the first 20 slowest modes which only represent 0.68% of the total modes, yet when combined they account for 11.08% of total motion of the protein. Similarly, the 8 non-degenerate modes only represent 0.27% of the total modes but account for 3.64% of total motion. The displacement vectors of the individual non-degenerate modes were studied as well as the MSF average over the 20 slowest and 20 fastest frequency normal modes.

**TABLE 1:** PTPS first 20 non-trivial normal modes, associated eigenvalues, and level of degeneracy.

Mode	Eigenvalue	Degeneracy	Contribution%
Mode 1	0.33	2	1.38
Mode 2	0.33	2	1.37
Mode 3	0.48	1	0.96
Mode 4	0.55	2	0.84
Mode 5	0.55	2	0.84
Mode 6	0.89	1	0.52
Mode 7	0.92	1	0.50
Mode 8	0.95	2	0.48
Mode 9	0.96	2	0.48
Mode 10	0.96	2	0.48
Mode 11	0.97	1	0.47
Mode 12	1.02	2	0.45
Mode 13	1.03	2	0.44
Mode 14	1.21	1	0.38
Mode 15	1.34	2	0.34
Mode 16	1.35	2	0.34
Mode 17	1.63	1	0.28
Mode 18	1.68	1	0.27
Mode 19	1.79	1	0.26
Mode 20	1.95	2	0.24

*The first six trivial modes were excluded, therefore mode 1 represents mode 7.*

**FIGURE 2:**
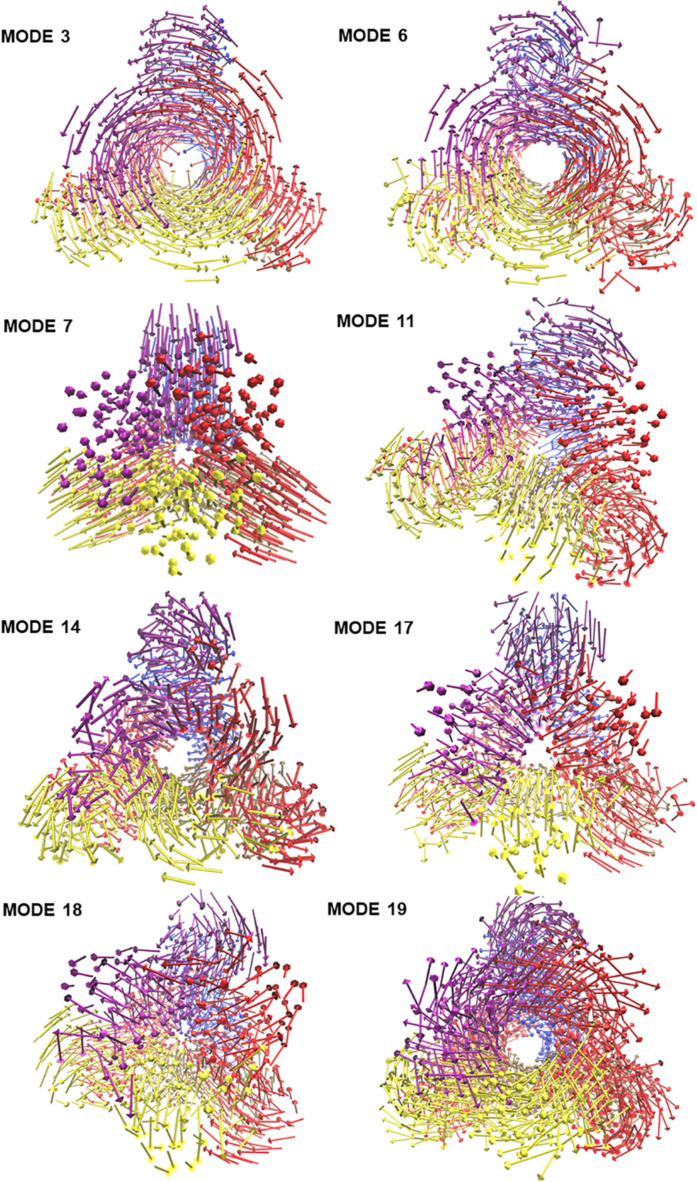
The projection of the eight slowest non-degenerate modes onto the structure of PTPS. Eigenvectors have been projected as a set of arrows that denote the direction of motion of the protein C_β_ atoms. The 3D structure is PTPS *P. falciparum* (PDB ID: 1Y13). Each of the protein chains is colored differently for illustration purposes.

To visualize the atomic displacements during the collective motions of PTPS, the respective eigenvectors of each of the eight non-degenerate modes were projected onto the structure of PTPS (Figure 2). Movies were constructed by projecting the eigenvectors onto the structure as a set of frames in which the vectors were added to the original atomic coordinates in increasing steps and then visualized using VMD. The atomic displacement during these modes was then examined to identify distinctive motions that are associated with the enzyme’s tunnel gating such as opening, closing or rotation as well as any other notable movements that resulted in structural changes around the tunnel or active site. The eight non-degenerate modes captured coupling movements between the tunnel gating, the N-terminal β-strands, and the central helices thus demonstrating that these regions primarily regulate the global dynamics of the protein. Furthermore, the observed structural changes of the tunnel were often accompanied by structural deformation around the active site region.

### PTPS Tunnel Displayed Several Distinct Movements That Involved the Fluctuations of the N-Terminal β-Strands

The modal analysis captured four distinctive motions that resulted in structural changes around the tunnel and the active site of PTPS. Mode 3 captured an asymmetric global twist of the terminal domains of the two PTPS trimers, characterized by their rotation in opposite directions (Supplementary Movie 1). From visual inspection, it appeared that the twisting of the terminal domains controlled the expansion and narrowing of the tunnel. Most notably, the diameter of the tunnel was smaller when the terminal domains followed a wringing motion with respect to the principal axis. We, therefore, suggest that the fluctuations of the terminal domains modulate the tunnel gating and consequently induces structural changes around the active site region. In mode 6, a notable tilt of the entire terminal domains was observed, leading to a side to side motion of the protein structure (Supplementary Movie 2). A stretching motion that resulted in the lateral expansion and contraction of the tunnel was captured in mode 7. From visual inspection, it appeared that this expansion and contraction was largely driven by the extensive movement of the terminal and central helices (Supplementary Movie 3). Mode 11 displayed a tilting motion of two central helices in an opposite direction to the third central helix (Supplementary Movie 4).

The identified motions within the tunnel were often accompanied by structural changes in the active site region, in which the tunnel acted as a connecting vessel to allow the entry of the substrate and provide the active site with flexibility. This further demonstrates how the tunnel gating mechanism exerts such control on the active site and illustrates the functional relevance of this cavity in the catalytic mechanism of the protein.

### The N-Terminal Domain Wringing Motion Modulated the Exposure of the Protein Tunnel

Visualization of the non-degenerate modes revealed three modes in which the wringing of the N-terminal domains promoted the surface exposure of buried regions within the tunnel. In particular, Mode 14 captured the outward and inward movement of the PTPS tunnel (likened to an engulfing movement) (Supplementary Movie 5). Mode 17 presented a prominent bending of the central helices from side to side, resulting in the protein tightening in the same direction (Supplementary Movie 6). Mode 18 featured a breathing motion that is characterized by the upward and downward movement of the central β-sheets (Supplementary Movie 7). Mode 19 displayed a clockwise rotation of the protein core that is associated with the terminal regions twisting in the opposite direction (Supplementary Movie 8). Within the eight identified non-degenerate modes, residue 76 to 114 of the N-terminal β-strands and residue 136 to 150 of the central helices exhibited the highest mobility (Figure 9A), which propelled the global motions of the protein. The expansion of the tunnel may promote the binding of PTPS to accessory proteins. Other proteins of this family are known to associate with other proteins, for example, the T-fold enzyme GCH1 is known to physically associate with another pentameric enzyme via the central opening of its tunnel in order to regulate its activity (Maita et al., 2004; Higgins and Gross, 2011).

Based on our modal analysis, we suggest that the flexibility of the tunnel is essential for the efficiency of the active site. The tunnel appears to modulate entry of the substrate via expansion and contraction which is primarily driven by the rotation of the N-terminal regions, thus allowing the substrate to pass through the tunnel into the active site for catalysis. Furthermore, we hypothesize that the twist and shear motions propel the substrate through the tunnel. Taken together, these results further the understanding of the functional dynamics of the PTPS enzyme, which will serve as significant guidelines toward the design of allosteric modulators that target structural regions that are pertinent to the function of the enzyme, and therefore the life cycle of the malarial parasite.

### MSF of the Individual Normal Modes Showed That the N-Terminal β-Strands and Central Helices Displayed the Highest Fluctuation

**FIGURE 3:**
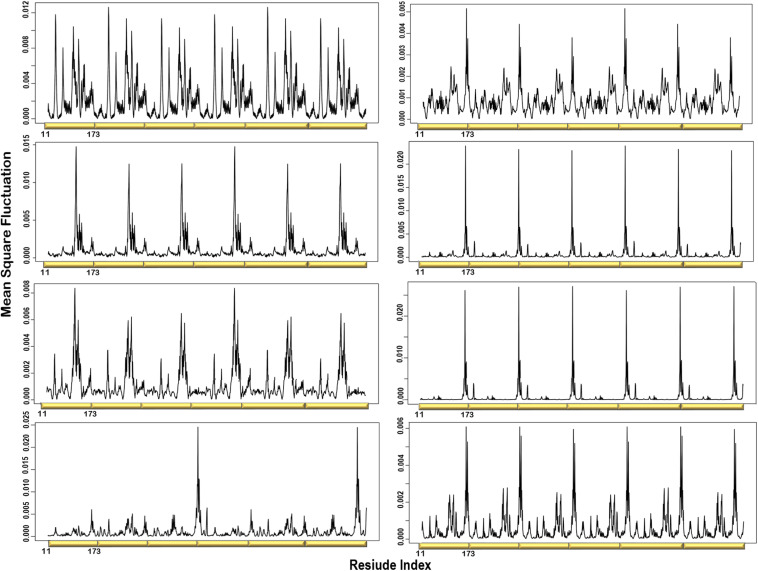
Individual MSF profiles of the eight non-degenerate modes. The yellow blocks represent each chain of the protein structure. The residues index is labeled for one chain along the lower abscissa and according to the *P. falciparum* (PDB ID: 1Y13) file.

**FIGURE 4:**
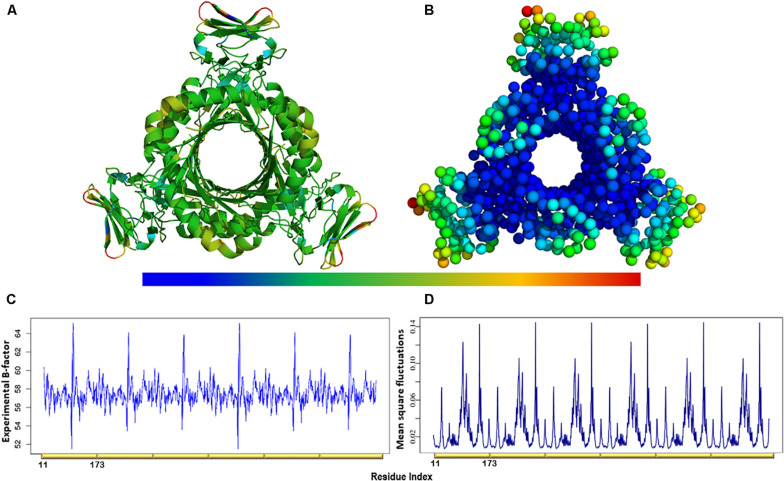
**(A)** The experimental B-factors mapped onto the PTPS 3D structure. **(B)** Atomic mobility over the slowest 20 modes. Regions of the lowest fluctuation are shown in blue, higher mobility in green, yellow, and red. The fluctuations color scale is from blue to red (High/flexible to low/rigid) atomic fluctuation. The figure demonstrates the high mobility of the terminal regions, central helices, and rigidity of the protein core, indicating that the NMA predicted motions have captured most of the experimentally determined motions. **(C)** Experimental B-factor plot and **(D)** Lowest 20 modes MSF profile. The residues index is labeled for one chain along the lower abscissa and according to the *P. falciparum* (PDB ID: 1Y13) file.

The atomic MSF profiles of the eight non-degenerate low-frequency modes were calculated and plotted (Figure 3). In each of the individual profiles, the N-terminal β-strands and the central helices have shown a notable fluctuation when compared to the protein core. Sharp peaks on the MSF profile distinguished these regions (Figure 3). The calculated MSF of the slowest 20 normal modes and the experimental B-factor were compared to validate the fluctuations that were predicted in the normal modes. Figure 4B shows the atomic fluctuation over the first 20 non-trivial low-frequency modes mapped onto the PTPS structure. The combination of the slowest 20 modes identifies the PTPS terminal domains as the most mobile (red) regions of the structure (Figure 4B). The simplified models of the ANM were shown to agree with the experimental B-factor results, as presented in Figure 4.

### Deformation Analysis Demonstrated a Significant Build-Up of Deformation Energy in the PTPS Tunnel

**FIGURE 5:**
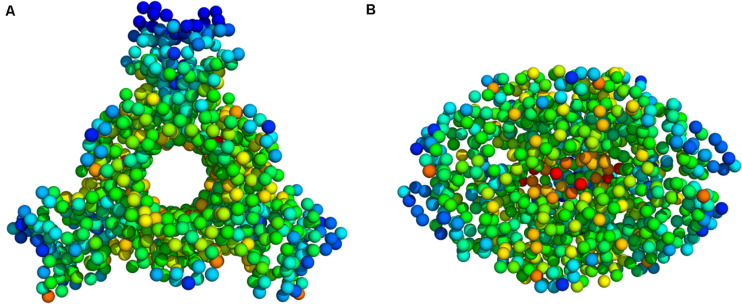
Deformation energy values mapped onto the PTPS 3D structure **(A)** Top view **(B)** Side view. The colors of the atoms indicate the amount of deformation. The dark blue regions are the least deformed, whereas red areas are strongly deformed.

Deformation is defined as any structural change caused by either an external force or a change in temperature (Bao, 2002). When the applied force is sufficient, a notable deformation can be observed; otherwise, the structure will resist the applied force and revert to its original state. After obtaining the normal modes, an analysis of the deformation energy distributed across the structure was performed to observe which regions of the structure were deformed during these global motions. The obtained results showed that the extensive fluctuations of the N-terminal domains, as seen in the MSF profiles, caused a significant build-up of deformation energy in the tunnel region of the enzyme. This demonstrates that the fluctuation of N-terminal domains has a long-distance effect on the tunnel core, which is located ∼30 Å away. The deformation energy values were calculated as the sum of the contributions of the first 20 non-trivial modes. The derived energy values were mapped onto the PTPS structure (Figure 5). We observed low deformation energy in the fluctuating terminal domains (blue), which was accompanied by high deformation energy within the tunnel region (red).

### Residue Cross-Correlation Analysis Presented the Movement of the N-Terminal Domains in a Concerted Manner

**FIGURE 6:**
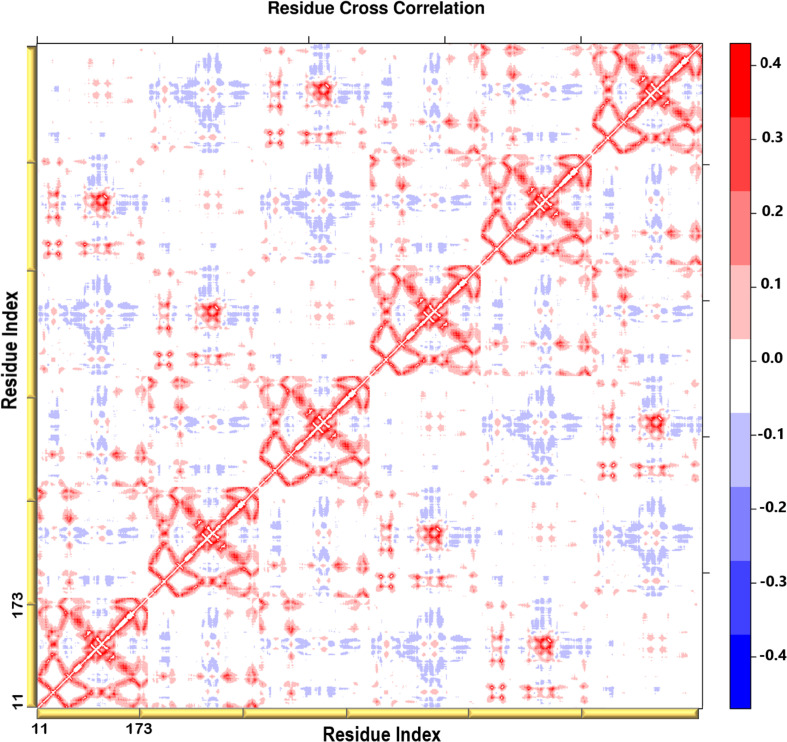
A heatmap representation showing the C_β_ atoms pairwise cross-correlation. The correlation matrix ranges from −0.4 to +0.4. The scale color bar on the right indicates the extent of the correlation in which the red color highlights correlated motions (residue pairs moving together in the same direction), while the blue color highlights the anti-correlated movements (residue pairs moving in opposite direction). The yellow bars along the bottom represent each chain of the protein.

A heatmap showing the cross-correlation of the C_β_ atom pairs is shown in Figure 6. Only motions in the first 20 non-trivial modes were selected in the calculation to highlight the residues involved in the protein collective motions. The residue cross-correlation analysis highlighted regions moving in a concerted manner, which therefore illustrates their involvement in the structural dynamics of the enzyme. The off-diagonal elements presented a positive correlation coefficient within the same chain, more specifically the residues of the terminal β-strands displayed correlation motion. Given the fact that PTPS is a multimeric protein, chains within one trimer exhibited a similar direction of motion. Anticorrelated motions were identified across the two dimers which can be understood due to the twisting or wringing of the helices across the trimers. The observed anticorrelation across the two trimers designates the opposing twisting or wringing of the terminals, therefore allowing the tunnel to open and close accordingly.

### Motif Analysis Revealed Uniquely Conserved Sites in Plasmodium PTPS Enzymes

Protein motifs are evolutionarily conserved sequence patterns that might unearth a biological function. Identifying the motifs and their structural locations can, therefore, provide insight into regions that modulate protein function. It can further reveal key residues that are unique and typically required to retain the protein function and structural stability (Mackenzie and Grigoryan, 2017; Ross et al., 2017; Zheng and Grigoryan, 2017).

**FIGURE 7:**
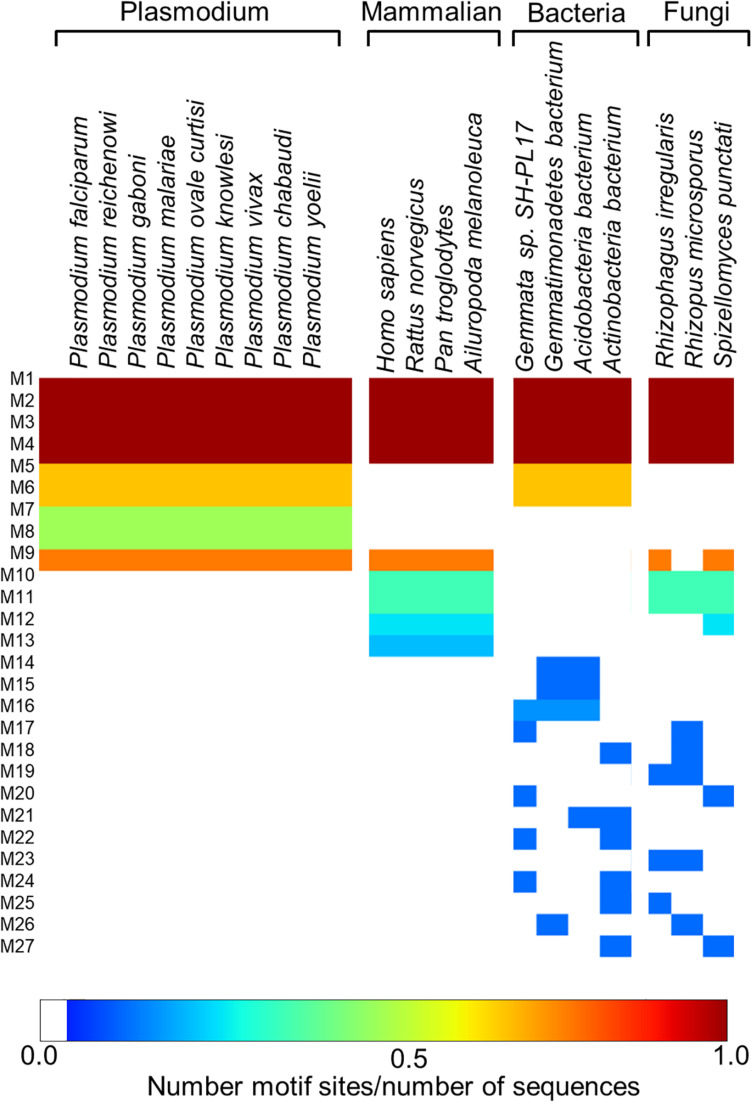
MEME heat map summarizing motif information for groups of PTPS homologue sequences. The white regions show sequences lacking a motif and the level of conservation increases from blue to red.

Following the analysis of the dynamics of the enzyme, we performed motif discovery to identify highly conserved motifs in *P. falciparum* PTPS and residues that are potentially involved in maintaining the secondary structure of the enzyme. The motif discovery was performed on the PTPS homologue sequences from four mammalian species including human, four bacterial species, three fungal species, and nine other Plasmodium species. A total of 27 motifs were identified. The analysis revealed four motifs (motifs 5–8) that were conserved in the Plasmodium PTPS enzyme sequences but were not detected in any of the mammalian species. Motifs 6–8 were uniquely conserved in the Plasmodium species, while motif 5 was also found among the bacterial and fungal PTPS enzymes. A heat map illustrating the occurrence of the motifs is shown in Figure 7.

**FIGURE 8:**
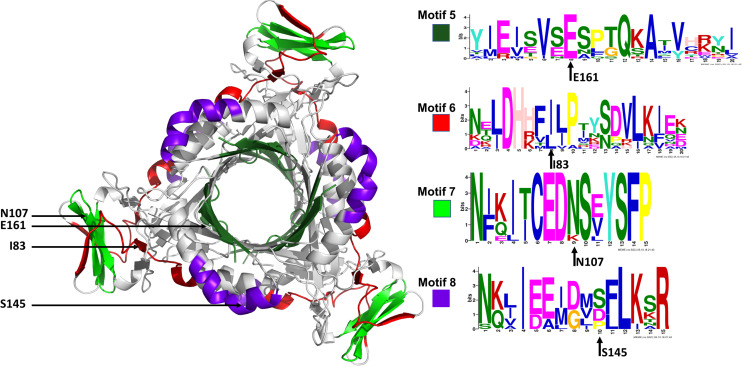
The MEME sequence logo contains a stack of letters at every position in the motif. The height of the letters represents the probability (in bits) of the letter occurring at that position multiplied by the number of times that residue occurs within that site in each motif site in the total dataset (Bailey et al., 2015).

The conserved Plasmodium motifs were mapped onto the crystal structure to identify their location (Figure 8). Motif 5 was located in the central β-sheet strands forming the tunnel cavity, motif 6 and 7 were located in the N-terminal antiparallel β-strands, as well as a loop linking the β-sheet strands and motif 8 was located in the central α-helices region. Motif 7 and 8 were shorter and more conserved relative to other motifs, which further suggests functional importance. As shown by the NMA, these regions were responsible for modulating the PTPS conformational transitions of the tunnel. The results presented significant structural and sequence differences between the Plasmodium PTPS enzyme and the human PTPS. As all of the identified motifs were not found in the human PTPS, this vital difference can be exploited for the attainment of drug selectivity and future antimalarial drug design.

### The Lowest and Highest-Frequency Normal Modes Revealed Residues of Notable Mobility That Were Also Located in the Conserved Structural Motifs

**FIGURE 9:**
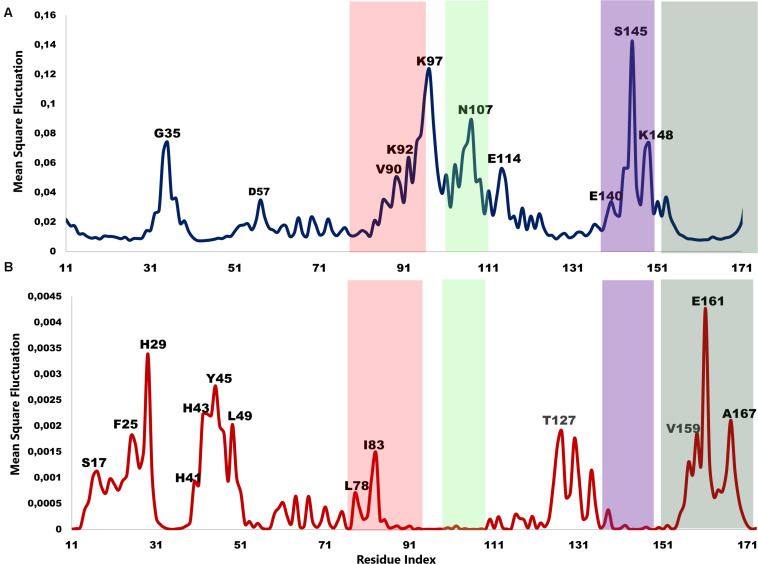
MSF profiles of **(A)** Low-frequency and **(B)** High-frequency normal modes. Residues with the highest fluctuations of the slowest modes and the fastest modes are labeled. The dark green, purple, red, and lime green bars highlight the location of motif 5, 6, 7, and 8 respectively. N107 is located in motif 7 (position 9), S145 in motif 8 (position 10), L78, and I83 are located in motif 6 (position 3 and 8) and V159, E161, and A167 are located in motif 5 (position 6, 8, and 14) respectively.

The low-frequency normal modes are often associated with large amplitude conformational changes which are essential for function (Mahajan and Sanejouand, 2015). The first 20 modes illustrate the substantial contribution of the PTPS helices to the protein’s global motion, in which they regularly control the tunnel movement via wringing or bending motions. In the MSF profile, the most substantial contributions to the atomic fluctuations emerged from the terminal and central helices (Figure 9A). The residues G35, K97, N107, and S145 showed notable fluctuations in the MSF profile. This demonstrates their key role in the global dynamics of the enzyme. Notably, residue N107 was found in motif 7 (position 9) and S145 was found in motif 8 (position 10) (Figure 8), further highlighting residues that are highly conserved in the Plasmodium species and which were active in modes that captured notable structural changes in the enzyme.

As the frequency increases, the modes become more localized and are accompanied by fast vibration of individual residues. It has previously been reported that high-frequency vibrating residues overlap with residues that are highly conserved and have an essential role in stabilizing the protein, and thus maintaining its function (Haliloglu et al., 2005). High-frequency vibrating residues in PTPS were identified from the average MSF calculated over the 20 highest frequency modes (Figure 9B). These included the active site residues H29, H41, and H43 as well as other important residues for the enzyme catalysis. The residues L78, I83, V159, E161, and A167 showed high fluctuation in the MSF profile of the fastest modes. Motif analysis showed that these residues do in fact overlap with sites of high conservation, with L78 and I83 found in motif 6 at (position 3 and 8), V159, E161, and A167 in motif 5 (position 6, 8, and 14), respectively (Figure 8).

**FIGURE 10:**
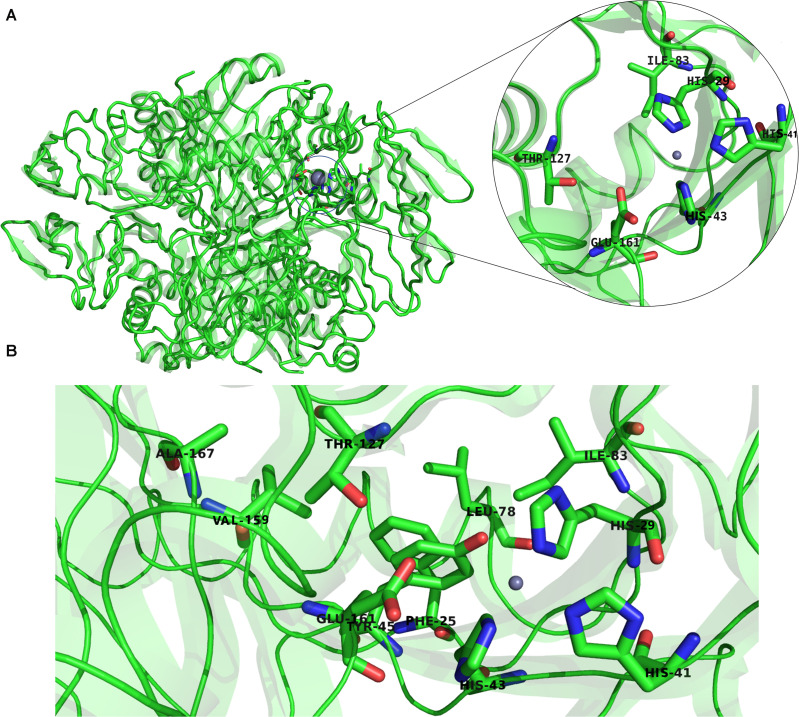
**(A)** The structure of *P. falciparum* PTPS and the metal center (active site) showing in the circled zoom with the active site residues: H29, H41, H43, and the two catalytically important residues T127 and E161. **(B)** Boxed zoom showing the location of the identified high vibrating residues.

Previous studies established that a cluster of hydrophobic residues surrounds the active site pocket and interacts with the substrate ring (Bürgisser et al., 1995; Colloc’h et al., 2000). Here we show that several of these hydrophobic residues also fluctuated in the high-frequency modes (Figure 10B). Residues E161 and T127, which are located at the bottom of the active site pocket (Figure 10A), showed significant fluctuation of the fast frequency modes, with E161 displaying the highest residue fluctuation (Figure 9B). These two residues were previously reported for their key role in substrate recognition and binding, as they both act as proton donors and acceptors during catalysis (Nar et al., 1994; Bürgisser et al., 1995; Ploom et al., 1999; Nar, 2011). Furthermore, Nar and colleagues reported that T105, T106, and E107, located around the active site pocket, constitute an acceptor site for the substrate ring during catalysis in the *Rattus norvegicus*. PTPS structure (PDB ID: 1B6Z). In the *P. falciparum* PTPS structure these residues are equivalent to S126, T127, E129, all three of which corresponded to high-frequency vibrating residues. Overall, the combined results obtained from our NMA and motif analysis have revealed conserved residues in the Plasmodium species that have key structural significance, with strong potential to modulate the stability and function of the enzyme. Thus, the characterized regions can then be proposed as alternatives that can be targeted and kept in consideration for future drug design efforts.

### Hinge Residues Were Identified and Found to Overlap With High-Frequency Vibrating Residues Located in the Protein Core

**FIGURE 11:**
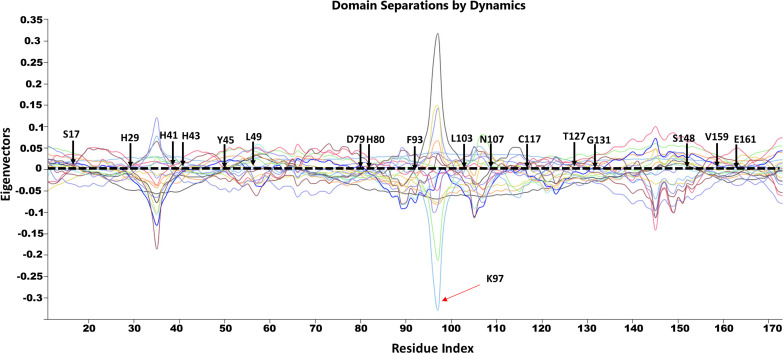
GNM based identification of global hinge sites over the low-frequency modes. Hinge residues are located at the crossover line where the eigenvectors’ values are equal to zero. Residues surrounding the hinge residues showed significant fluctuation in the positive and negative direction of motion. The identified hinge residues include the active site residues, H29, H41 and H43; the catalytically important residues, H80, T127 and E161; and the active site neighboring residues, Y45, L49, D89, F93, L103, N107, C117, S148, V159 which are illustrated by arrows.

Hinge regions are often found between domains in a protein to allow flexibility, in which they permit the domains to move relative to one another or clamp down on a substrate (Towler et al., 2004; Yang and Bahar, 2005; Amusengeri and Tastan Bishop, 2019). The hinge residues, act as anchoring points and are involved in the propagation of large-scale conformational changes. The ANM provides a 3D description of motions identified in the modes, whereas in GNM the motion is projected to a mode space of N dimensions and therefore provides a description of atoms’ mean squared displacements. GNM calculations are considered to be the preferred method for predicting the magnitude of motions at the cost of losing directions (Atilgan et al., 2001) and thus easily allow the identification of hinge sites. The DynOmics portal was used to recognize hinge residues (Li et al., 2017). The server provided information about key sites involved in the collective mechanics and allostery of the protein by mapping of the coarse-grained conformations driven by collective modes to their full-atomic representations. The domain separations analysis based on modes of the GNM disclosed hinge sites (residues that exhibit minimal displacements in the softest two modes). The labeled residues in Figure 11 correspond to the interfacial residues whose neighboring residues have a different sign in the eigenvector relative to themselves. Thus, these interfacial residues act as hinges about which their neighboring residues are displaced in opposite directions. Specifically, we identified hinge sites that overlapped with residues of the active site: H29, H41, and H43 as well as E161. The functional importance of these residues was supported by previous studies in which the mutation of these residues resulted in either a complete or a dramatic loss of enzymatic activity (Bürgisser et al., 1995; Nar, 2011). Furthermore, the active residues were responsible for the coordination of the catalytically important metal ion (Khairallah et al., 2020). The residues Y45, L49, T127, S148, V159, E161 (as well as their neighboring residues) also corresponded to high frequency vibrating residues that were identified in the ANM (Figure 9B), suggesting that high-frequency vibrating residues correspond to rigid points about which significant conformational changes occur.

**FIGURE 12:**
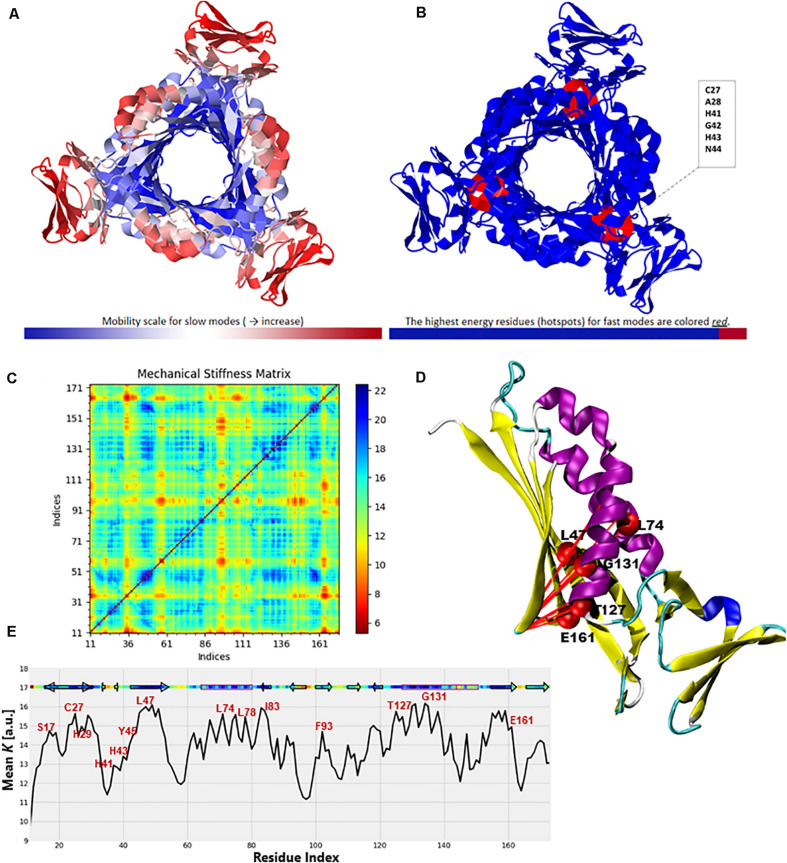
Color-coded representation of the PTPS structure based on the mobility of the residues within the **(A)** Low-frequency and **(B)** High-frequency GNM modes. The color scale varies from blue as the most rigid to red as most mobile sites. The figures were obtained from the DynOmics portal (Li et al., 2017). **(C)** A mechanical resistance matrix obtained by calculating the effective force constant in response to uniaxial extensional forces exerted at each pair of residues. The scale varies from blue as residue pairs of the strongest interaction/rigid to red residue pairs of the weakest interaction/flexible. **(D)** A cartoon representation of a single chain of *P. falciparum* PTPS with the residues of strongest interaction mapped onto the structure and shown as red spheres. **(E)** The mean value of the effective spring constant over all pairs for each residue, with the secondary structures shown along the upper abscissa. The color bar shows strong regions/rigid in blue and weak regions/mobile in red. The figures were generated using the Matplotlib library and VMD.

To further validate the high-frequency vibrating residues that were identified in the ANM, we used the DynOmics portal to analyze the mechanical properties of PTPS to identify the most rigid residues in the GNM (Figure 12). The results obtained from the DynOmics portal were in agreement with our findings from the ANM. Firstly, the N-terminal and central helices were predicted to have high mobility in the low-frequency modes, while the most rigid residues were located in the active site region (Figure 12A). Secondly, the GNM calculations performed using DynOmics located high energy hotspots in the active site region which directly overlapped with the high-frequency vibrating residues that were identified in the ANM (Figure 12B).

The ProDy Python package for protein structural dynamics analysis (Bakan et al., 2011, 2014) was also used, in particular, the ProDy MechStiff function to evaluate the mechanical stiffness of the PTPS protein (Eyal and Bahar, 2008). A mechanical stiffness matrix was then produced by calculating the effective force constants in response to uniaxial extensional forces exerted at each pair of residues (Eyal and Bahar, 2008; Figure 12C). The obtained results illustrated that pairs located in the active site and the surrounding region exhibited strong pairs of interactions. This suggests that the residues of the active site H29, H41 and H43 as well as its surrounding catalytic residues including T127 and E161 bear a relatively strong resistance to deformation. Figure 12E shows the results averaged over all pairs for each residue, which provides a profile of the mechanical resistance of individual residues to deformation. Some residues, especially those located in the active site and surrounding area were more rigid than others and more resistant to deformation, as indicated by this profile. More specifically, these residues involved the active site residues H29, H41, and H43 as well as S17, L78, I83, F93, T127, G131, and E161 which were also among the highly conserved, high frequency vibrating and hinge residues. A cartoon representation of a single chain of the *P. falciparum* PTPS is shown in Figure 12D. The figure shows pairs of residues with the strongest effective force constant and their location within the protein structure. The identified pairs of T127, E16, G131 were located around the active site area and were also among the high vibrating and hinge residues.

## Conclusion

Antifolate drug resistance is a major challenge in the fight against malaria and the need to develop new drugs with a unique mechanism of action has become more crucial than ever. In this study, we classify the dynamics of the parasite’s *de novo* folate synthesis pathway enzyme PTPS, in an attempt to uncover key regions that modulate conformational transitions which are imperative to its function. Notable global motions of functional significance were captured within the low-frequency non-degenerate modes. In particular, we showed that the opening and closing of the PTPS central tunnel was driven by the distinctive twisting and wringing of the terminal regions. Furthermore, the displacements observed in the PTPS N-terminal domains appeared to have a long-distance regulatory effect on the rigid core of the protein, located more than 30 Å away. Motif analysis further validated our findings with the identification of structural motifs that are uniquely conserved in the Plasmodium PTPS enzymes. These conserved motifs were located in the N-terminal domains, the central helices as well as the protein tunnel, and point toward the functional importance of these regions in the Plasmodium PTPS enzyme. Collectively these results suggest an opportunity for selective inhibition as these regions are not conserved in Human PTPS. Specifically, these regions can be proposed as potential allosteric sites for future antimalarial drug discovery attempts.

## Data Availability Statement

All datasets presented in this study are included in the article/Supplementary Material.

## Author Contributions

ÖTB conceived the project. AK did the calculations and analysis of data under the supervision of CR and ÖTB. AK wrote the first draft. All authors edited it to the final version.

## Conflict of Interest

The authors declare that the research was conducted in the absence of any commercial or financial relationships that could be construed as a potential conflict of interest.
