# Supplementary material for: Probing the Structural Dynamics of the Plasmodium falciparum Tunneling-Fold Enzyme 6-Pyruvoyl Tetrahydropterin Synthase to Reveal Allosteric Drug Targeting Sites
Source: Front Mol Biosci. 2020 Sep 25;7:575196. doi: 10.3389/fmolb.2020.575196 (PMC7546909; doi:10.3389/fmolb.2020.575196)
Supplement: Supplementary file 1 [file Table_1.DOCX]

***Supplementary Material***

**Supplementary Table 1.** Organism names and accession numbers the 6-pyruvol tetrahydropterin synthase amino acid sequences used in this study.

| **Species** | **Accession Number** |
| --- | --- |
| ***Plasmodium falciparum* 3D7** | **PF3D7_0628000** |
| ***Plasmodium reichenowi* CDC** | **PRCDC_0626400** |
| ***Plasmodium gaboni* G01** | **PGABG01_0626800** |
| ***Plasmodium malariae* UG01** | **PmUG01_11035600** |
| ***Plasmodium ovale curtisi* GH01** | **PocGH01_11029300** |
| ***Plasmodium knowlesi* H** | **PKNH_1121800** |
| ***Plasmodium vivax* P01** | **PVP01_1121500** |
| ***Plasmodium chabaudi chabaudi*** | **PCHAS_1126200** |
| ***Plasmodium yoelii yoelii* 17X** | **PY17X_1128200** |
| ***Homo sapiens* (Human)** | **Q03393** |
| ***Rattus norvegicus* (Rat)** | **P27213** |
| ***Pan troglodytes* (Chimpanzee)** | **H2QB30** |
| ***Ailuropoda melanoleuca* (Giant panda)** | **D2GYP9** |
| ***Gemmata* sp. SH-PL17** | **A0A142XE24** |
| ***Gemmatimonadetes bacterium*** | **A0A2V7MAP3** |
| ***Acidobacteria bacterium*** | **A0A2V8BFN7** |
| ***Actinobacteria bacterium*** | **A0A2M7TB79** |
| ***Rhizophagus irregularis*** | **A0A2I1GC34** |
| ***Rhizopus microsporus*** | **A0A0C7CEB1** |
| ***Spizellomyces punctatus*** | **A0A0L0HI22** |
